# From Sensor-Empowered Ubiquitous Computing to Embodied Intelligence: Architectures, Paradigm Evolution, and Emerging Challenges

**DOI:** 10.3390/s26144352

**Published:** 2026-07-09

**Authors:** Ali Jia, Ziwei Cai, Xiaoyuan Liu, Kechen Zheng, Jia Liu

**Affiliations:** 1 Shanxi Province Intelligent Optoelectronic Sensing Application Technology Innovation Center, Yuncheng University, Yuncheng 044000, China; jiaali@ycu.edu.cn; 2 School of Mathematics and Information Technology, Yuncheng University, Yuncheng 044000, China; 3 Beijing Petroleum Machinery Co., Ltd., Beijing 102206, China; liuxiaoydr@cnpc.com.cn; 4 School of Computer Science and Technology, Zhejiang University of Technology, Hangzhou 310014, China; kechenzheng@zjut.edu.cn; 5 Center for Strategic Cyber Resilience Research and Development, National Institute of Informatics, Tokyo 101-8430, Japan; jliu@nii.ac.jp

**Keywords:** ubiquitous computing, embodied intelligence, edge intelligence, multi-modal perception

## Abstract

With the rapid development of artificial intelligence technology, the transportation, industry, and healthcare fields are undergoing an intelligent evolution. These advancements have raised higher requirements for technologies such as mobile robots, wearable intelligent agents, self-driving cars, and unmanned aerial vehicles. Compared with traditional discrete sensor architectures, highly integrated sensing systems deliver superior speed, efficiency, and reliability to satisfy the stringent requirements of emerging intelligent devices. By integrating advanced technologies such as perception, communication, and computing, the process of system intelligence is accelerating, driving us into the era of embodied intelligence. Thus, sensors are no longer merely passive data collection tools but have transformed into core components that drive the connection between perception and action. To help researchers better understand this transformation and clarify the implementation path, we summarize the key technological advancements in related fields. Firstly, we review the related technological developments, including the sensor, multi-modal perception, wireless communication, and edge computing technology. Then, we explore the limitations of traditional sensors and independent computing models, especially the trade-offs among latency, energy efficiency, and system reliability. Subsequently, we introduce innovative technologies that drive the development of embodied intelligence, covering advanced learning mechanisms such as multi-agent systems, reinforcement learning, and federated learning. Finally, we compare the typical application scenarios of the two paradigms and discuss the challenges faced by existing technologies and standardization. We also look forward to future research directions in this field.

## 1. Introduction

Embedded computing and ubiquitous computing are constantly evolving in their respective fields, continuously providing users with computing and communication services, and playing significant roles in daily life, industrial production, and social activities [1]. Unlike traditional computing models, pervasive computing integrates technologies from multiple fields such as embedded systems, wireless networks, mobile computing, and distributed computing. This integration breaks through the boundaries between people and devices, as well as between the digital space and the physical world. To enable software systems to model and perceive the physical environment, dedicated sensing hardware is required to capture real-world data and contextual information. As the main interface for the system to perceive the physical world, sensors are widely used in environmental data collection, intelligent decision-making, real-time response, etc. [2]. Therefore, the performance and capability of sensors directly affect the functionality, efficiency, and intelligence level of these systems.

Over the past few decades, sensor technology has developed rapidly. Both the academic and industrial sectors have conducted in-depth research on sensor miniaturization, low power consumption, and multi-modal fusion, and significant progress has been made in embedded and pervasive computing systems. With the continuous growth in demand in application fields such as smart homes, mobile health, and industrial Internet, these systems have been further promoted [3]. The miniaturization and intelligence process of sensor systems has been accelerated through the deep integration of perception modules and computing modules [4]. This progress is driving sensors to be better integrated into embedded devices and ubiquitous computing frameworks. In the smart home scenario, automation has become a typical example of ubiquitous computing penetrating daily life. On-site programming technology enhances the adaptability of sensor systems in home environments [5]. In the field of mobile health, deep-learning-based multi-modal sensor fusion technology has significantly enhanced the accuracy and reliability of wearable health monitoring devices. This development has driven the growth of ubiquitous computing applications [6]. In the field of the industrial Internet, efficient visual perception and fault detection technologies have promoted the integration of ubiquitous computing with industrial production. These technologies are addressing key application bottlenecks [7].

The deep integration of emerging communication and computing technologies with sensor systems has become a key research direction in both academia and industry. Researchers are committed to addressing key challenges such as the collaborative optimization of overall system performance and practical application bottlenecks. AI-based integrated perception and communication technology provides a new solution to overcome the performance limitations of traditional sensor systems [8]. Traditional sensor-driven ubiquitous computing systems typically follow an open processing chain: “perception-reporting-response”. However, this method has inherent limitations. It is difficult to support long-term autonomous operation, goal-driven decision-making, and complex physical interactions. Recent studies have extensively analyzed these limitations and proposed solutions for the transition to embodied intelligence (EI). EI needs to be achieved through a closed-loop structure of “perception-thinking-action”. This requires the system not only to actively perceive but also to learn goal-oriented behaviors and continuously interact with the physical environment [9]. The currently feasible solution is to enhance the perception ability of embodied agents in three-dimensional physical environments through multi-modal perception fusion technology, thereby promoting the transition from ubiquitous computing to embodied intelligence.

It is important to clarify the relationship between ubiquitous computing [1,10], ambient intelligence [11], and embodied intelligence. The inherent limitations of ambient intelligence stem from the technical bottlenecks of early sensors and computing architectures. Traditional sensors only support passive data collection, and early ubiquitous computing platforms lack powerful on-site computing resources and closed-loop control frameworks. Under such conditions, systems can only implement environmental perception and preset passive responses, and cannot actively interact with physical environments. The emergence of 6G communication, mobile edge computing, and embodied learning has fundamentally broken these constraints. Specifically, 6G provides ultra-low-latency synchronous transmission for perception-action links. Edge computing migrates computing power to terminal nodes to support real-time local decision-making. Embodied learning enables systems to accumulate experience through continuous interaction. These technologies jointly drive the paradigm shift from passive ambient intelligence to goal-driven, interactive embodied intelligence.

### 1.1. Research Gaps and Research Motivation

To date, although researchers have conducted extensive studies in the fields of embedded computing and pervasive computing for many years, there are still many key issues. More importantly, the evolution from traditional ubiquitous computing to embodied intelligence also brings many challenges. The integration of emerging wireless technologies and low-power wide area networks (LPWAN) is facing some bottlenecks, which have led to slow progress in the development of low-power embedded intelligence [12,13]. On the other hand, the amount of data generated by sensors is increasing rapidly. The heterogeneity in the data collection process, communication load, limited storage space, and the limitations of analysis methods are all affecting the intelligence of the system. The demand for continuous learning from embodied intelligence has further intensified the need for efficient data management systems [14]. The issues of security, reliability, and credibility in sensor networks have received increasing attention. In embodied intelligent systems that directly interact with the physical world, malicious attacks such as data poisoning and perceptual deception pose serious risks. These attacks not only reduce system performance but also pose significant physical security risks, including covert poisoning attacks on recognition models and perceived spoofing threats that lack effective defense strategies [15,16]. The application of machine learning, edge computing, and fog computing in sensor systems is still in the exploratory stage. The deep integration of these technologies with the embodied intelligence closed-loop architecture faces multiple challenges, with key issues including lightweight model design, dynamic resource allocation, and real-time decision-making [17]. The majority of existing review articles only focus on a single field, such as sensor-driven ubiquitous computing or embodied intelligence. These articles fail to systematically analyze the evolutionary relationship between the two, especially the key technologies that drove this transformation.

To fill these research gaps, this paper aims to systematically summarize the latest research progress in the field of sensor-driven embedded and pervasive computing. The content covers analysis, design, optimization, implementation, and standardization, with a particular focus on the technical paths for the transition to embodied intelligence. By integrating scattered research results, we clarify the intrinsic connection between pervasive computing and embodied intelligence. It highlights the crucial role of sensors in this paradigm shift. In addition, we also identify the key directions for future research. Through this comprehensive overview, this article hopes to provide researchers and practitioners with a systematic reference framework to promote further development in these fields.

### 1.2. Research Scope and Main Contributions

Table 1 compares sensor-driven ubiquitous computing and embodied intelligence systems across five core dimensions: design goals, decision loops, learning modes, system architectures, key constraints, and failure consequences. Each row highlights the paradigm shift from passive, data-centric, open-loop ubiquitous computing to goal-driven, closed-loop, physically interactive embodied intelligence.

This paper focuses on the complete evolution from sensor-driven embedded and pervasive computing to embodied intelligence. We systematically cover all research stages, from core supporting technologies to application scenarios. We discuss fundamental technologies such as sensor integration, wireless communication, and data management. We analyze the limitations of traditional ubiquitous computing and discuss the key technological breakthroughs that drive the development of embodied intelligence. In addition, we also examine the current challenges and propose possible solutions.

The main contributions of this paper can be summarized as follows:

- To overcome the challenges of integrating sensing technology with emerging communication, computing, and learning paradigms, we have delved deeply into the integration and optimization of these technologies. We focus on the core role of sensors and summarize the key technological breakthroughs that drive the transformation from ubiquitous computing to embodied intelligence.
- To address the need for a comprehensive comparison between sensor-driven systems and embodied intelligence, we analyze the typical application scenarios of both. We highlighted their value in practical applications and compared the differences between the two paradigms in terms of goals, performance requirements, and design constraints.
- To better understand the research challenges encountered during the evolution process, we have identified the main issues. We have put forward the research frontiers for the future of embodied intelligence and provided valuable guidance and reference for subsequent studies.
- Different from prior surveys that separately discuss ubiquitous computing or embodied intelligence in isolation, this review constructs an integrated evolutionary framework linking the two paradigms. By tracing the technical transition path from sensor-enabled pervasive systems to embodied agents, this review provides a holistic cross-domain analytical perspective absent from previous literature.

### 1.3. Review Structure

**Figure 1:**
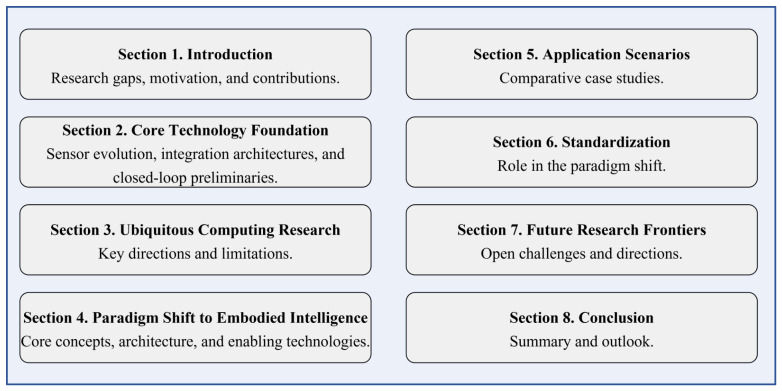
The structure of this paper.

The remainder of this paper is organized as follows, as shown in Figure 1. In the second section, we introduce the core technology foundation. We discuss the evolution of sensor technology, the integration architecture of sensors with embedded and pervasive computing, and the initial exploration of closed-loop systems. These contents lay the foundation for achieving embodied intelligence. In the third section, we focus on the key research directions of sensor-driven embedded and pervasive computing. We discuss wireless technology integration, data management, machine learning applications, security, and spectrum management. Meanwhile, we also analyze the limitations of these directions in supporting autonomous physical interaction. In the fourth section, we discuss in detail the transition from sensor-driven ubiquitous computing to embodied intelligence. We explore the core concept of embodied intelligence, the upgrade of sensor roles, and the optimization of closed-loop architectures. We also emphasize the key technologies that drive this paradigm shift. In the fifth section, we introduce typical application scenarios and case studies. We compare the performance and applicability of sensor-driven ubiquitous computing and embodied intelligence in different fields. In the sixth section, we examine the role of standardization work in promoting the transformation from sensor-driven ubiquitous computing to embodied intelligence. In the seventh section, we look forward to the future research frontiers of sensor technology, ubiquitous computing, and embodied intelligence integration development. Finally, in the eighth section, we summarize the entire paper and emphasize the significance and future prospects of the evolution from sensor-driven ubiquitous computing to embodied intelligence.

## 2. Evolution of Sensor-Empowered Ubiquitous Computing and Preliminaries for Embodied Intelligence

The conceptual foundation of sensor-empowered ubiquitous computing originates from Mark Weiser’s visionary work, which defined ubiquitous computing as “the third wave of computing” beyond mainframes and personal computers [10,18]. Weiser envisioned computers that “disappear into the environment,” supporting silent collaboration and adapting to human activities without explicit interaction. Evaluating progress against his original goals, we find that several aspects have been achieved, including miniaturized computing nodes, pervasive wireless networks, distributed sensors, and context awareness. Some goals are partially fulfilled, such as adaptive environments, smart homes, wearable computing, and basic IoT automation. Others remain unfulfilled, including true autonomous physical interaction, lifelong learning, goal-driven closed-loop behavior, and seamless human–machine–environment symbiosis.

The development of sensor-driven embedded and ubiquitous computing depends heavily on our advancements in sensor technology. We also prioritize the efficient integration of sensors with computing, communication, and networking systems. Meanwhile, our research into closed-loop systems, autonomous learning, and physical interaction in ubiquitous computing is laying a strong foundation for the transition to embodied intelligence. In this section, we focus on the development of sensor technology and its integration architecture. We also cover key supporting technologies and explore preliminary work on closed-loop systems. The analyses laid out herein establish the theoretical and technical foundations for the subsequent discussion of the paradigm shift toward embodied intelligence.

### 2.1. Evolution of Sensor Technology in Embedded and Ubiquitous Computing

Sensor technology has gone through three key evolutionary stages to adapt to the development of embedded and pervasive computing. These continuous advancements laid the foundation for the emergence of embodied intelligence [19]. In the early stage, sensors were mainly single-mode, bulky, and energy-consuming. They existed as peripheral components for specific data acquisition tasks, such as temperature sensors in industrial control systems. These sensors had limited functions, poor compatibility, and were unable to support complex perception or interaction, let alone meet the demands of embodied intelligence [20]. In [21], Goumopoulos et al. proposed a high-precision temperature measurement system, which achieved a measurement accuracy of ±0.03 °C within the range of 5–45 °C by adopting negative temperature coefficient thermistors and advanced calibration methods. In [22], Li et al. discussed the design of an interleaved capacitive humidity sensor and proposed an optimized scheme that balances dynamic response and sensitivity, thereby achieving a measurement accuracy exceeding 95%. Furthermore, Han et al. [23] proposed a novel outdoor air quality monitoring system that utilizes two methods to enhance the prediction quality. On the one hand, the system ensures the accuracy of pollution data collection through the Zigbee network. On the other hand, an improved long short-term memory model is designed to predict the pollution cycle.

In recent years, micro-electromechanical systems (MEMS) technology has made significant progress, and as a result, sensors have gradually entered a stage of miniaturization and low power consumption. MEMS technology enables sensors to be conveniently integrated into small embedded devices, especially in the miniaturization applications of smartphones and wearable devices. These miniaturized devices make the perception system ubiquitous, thus enabling the collection of a broader range of data about the physical world. In [24], Zhu et al. summarized the main progress of MEMS technology, analyzed the transformation from rigid MEMS to flexible MEMS, and emphasized the key role of this change in wearable systems. In [25], Chircov et al. discussed MEMS in the integration of mechanical and electrical components of the research development of precision equipment, and summarized its energy acquisition and its wide use in the field of environmental monitoring. In [26], Shah et al. reviewed the design challenges of MEMS microphones, covering design issues such as high sensitivity, flat frequency response, and low noise.

In the field of biomedicine, the advancement of sensor technology has driven the development of applications such as drug synthesis, minimally invasive surgery, and diagnosis. Ref. [27] proposed an ultra-low-power mixed-signal system-on-chip for multi-source energy harvesting and wireless power transmission. The researchers adopted intelligent power management and RF-to-DC converters in this system. This system provides power to wireless sensor nodes and supports multiple energy sources and applications. This development marks a significant advancement in the sustainability and multi-functionality of sensor systems in real-world environments. With the continuous development of sensor technology, Li et al. [28] pointed out that multi-modal integration has become a common trend. This trend provides a foundation for environmental perception and situational awareness. It also plays a key role in autonomous decision-making in embodied intelligence. However, at this stage, sensors still lack active adaptability. They are limited to passive perception and can only respond to environmental changes [29].

Due to the demands of diverse applications, sensor technology has been evolving towards multi-modal, intelligent, networked, and active perception [30]. Regarding multi-modality, in [31], the authors integrated multiple types of information into multi-modal sensors, including visual, auditory, mechanical, positional, and environmental perception. By integrating these inputs, a comprehensive collection of environmental data has been achieved, significantly enhancing the accuracy and robustness of perception. In [32], Chatterjee et al. studied how to equip intelligent sensors with basic computing and signal processing capabilities, thereby reducing the data transmission overhead of the system and the burden of centralized computing. Furthermore, in [33], Fascista et al. proposed a framework for networked sensors, constructing a distributed sensor network through wireless interconnection to achieve large-scale and real-time environmental monitoring over a wide area.

### 2.2. Sensor Integration Architecture in Embedded and Ubiquitous Computing

In this paper, we include integrated sensor systems in this review because they form the essential foundation for the paradigm shift from sensor-driven ubiquitous computing to embodied intelligence. A strong integration of sensors, computing, and communication is a prerequisite for achieving closed-loop embodied intelligence. Ubiquitous computing embeds computing power into devices, enabling these devices to communicate with each other and perform tasks independently without interaction. In addition, the integrated use of location information and activity detection technology enables devices to be more intelligent and better adapted to the environment, thereby promoting the wide application of ubiquitous computing. As the authors discussed in [34], an adaptive service system has been implemented through dynamic and context-aware applications. In addition, in [35], Suciu et al. explored the application of ubiquitous computing in industry and scientific research, and investigated the mobility of wireless sensor networks (WSNs). Meanwhile, in [36], Dhyani et al. summarized pervasive computing’s key challenges, including hardware limits, security risks, power consumption, cost, and connectivity issues.

Wearable devices are considered typical examples of sensor-integrated ubiquitous computing systems in the early stages of evolution, rather than fully developed embodied intelligence systems. In [37], Bayo-Monton et al. proposed a wearable hardware framework integrating physiological sensors with low-cost microcontrollers (Arduino, Raspberry Pi) to mitigate transmission delay and data loss. By enhancing the effectiveness of biological signal transmission and analysis, this scheme can be further expanded into a telemedicine system. In [38], Mekruksavanich et al. proposed a framework for human activity recognition based on deep learning through integrating three-axis gyroscope and accelerometer data to realize user identity identification.

In the field of communication, the authors of [39] proposed a new channel equalization method, aiming to address the common “channel collapse” problem in traditional deep learning models. They adopted de-correlation and whitening techniques. These operations have significantly enhanced the performance of multi-modal human activity recognition and also strengthened the robustness of wearable sensor networks in real-time applications. In addition, the authors of [40] proposed a lightweight and scalable blockchain framework named “Sensor-Chain”. This framework is designed to address the challenges of data collection, storage, and analysis in the Internet of Things. Through this framework, the resource consumption of the system is reduced while ensuring the integrity of data. By combining sensors with cloud computing and intelligent data processing, BSNs can improve the quality of nursing, reduce medical costs, and improve the health of patients [41].

In embedded and pervasive computing systems, researchers typically adopt a hierarchical architecture for sensor integration. This architecture mainly consists of four layers, including the perception layer, the network layer, the computing layer, and the application layer. It ensures seamless data flow from data collection to practical application. However, although this architecture supports efficient perception and some basic intelligent responses, it still has some limitations. These limitations prevent it from fully supporting autonomous interaction. The perception layer includes various types of sensors, signal conditioning modules, and data acquisition systems, which are used to collect data from the physical world. First of all, it collects diverse environmental data, such as temperature, humidity, sound, images, etc. Then, the collected analog signals are converted into a binary format that the computing system can process. Based on this, basic processing of the signal is carried out, including noise removal and filtering, etc., to improve data quality [42]. In contrast, the network layer is responsible for data transmission tasks, transmitting preprocessed data to the computing layer. The network layer involves modern wireless communication technologies such as Wi-Fi, cellular networks or LoRa, etc., and requires these networks to meet diverse demands such as latency, bandwidth, and energy efficiency. The functions of the computing layer include data storage, analysis, and intelligent decision-making. To meet the requirements of computing efficiency and real-time performance, edge nodes usually handle real-time computing, while cloud nodes are responsible for large-scale data storage and complex model training [43]. The application layer, as the user-facing interface, provides personalized services based on sensor data and computing results [44].

Traditional hierarchical architectures are effective in practical applications, but they are essentially data-driven rather than goal-driven. Loquercio et al. [45] point out that the coupling between the perception layer and the application (or action) layer is relatively weak. Therefore, there is a lack of closed-loop interaction between perception and action, which limits the ability to make real-time autonomous decisions. In these architectures, the integration of perception, action, and feedback loops makes true autonomous systems possible [46].

### 2.3. Basic Technologies and Embodied Intelligence Enablers

Ubiquitous computing is a paradigm that embeds computing and sensing capabilities into everyday environments and devices, enabling passive perception, data collection, and human-centered reactive services through an open-loop architecture. Within this broader paradigm, embedded computing refers to dedicated computing systems integrated into devices or machines, focusing on specific functions, real-time performance, and hardware–software co-design. In contrast, pervasive computing emphasizes computing resources seamlessly integrated into everyday environments, providing invisible, context-aware, and human-centered intelligent services. The core distinction lies in the fact that embedded computing targets standalone, task-specific intelligent devices, whereas pervasive computing focuses on interconnected, environment-embedded distributed systems. The smooth operation of sensor-driven embedded and pervasive computing systems depends on the low-power technology and resource allocation. These technologies form the foundation of the development of ubiquitous computing and play a significant role in promoting the transformation towards embodied intelligence [47].

Researchers have made significant progress in low-power technology, especially in the field of battery-powered sensor nodes. In [48], Karthick et al. designed a low-power sensor that integrates solar energy acquisition technology and optimizes power management to extend the system’s service life. Embedded devices such as robots and wearable devices, due to energy constraints, are often difficult to use for long periods of time, which is also one of the main research directions at present. For instance, in [49], Pagan et al. introduced an ultra-low power activity identification system that significantly reduces transmission overhead and thereby lowers energy consumption by adopting adaptive compressive sensing technology. A single technology often fails to achieve the best energy-saving effect. Therefore, the researchers also optimized the sensor positioning and unsupervised clustering modules, further enhancing energy efficiency. In [50], Nurelmadina et al. explored the application of LPWAN technology in the Industrial Internet of Things (IIoT), aiming to reduce the operational energy consumption of sensors by rationally allocating network resources. Similarly, in [51], Chen et al. proposed a cognitive LPWAN architecture that combines cognitive radio technology to ensure the reliability and energy efficiency of low-power systems.

When sensors, communication transceivers, and computing resources are integrated, especially when a network gradually forms, reasonable resource allocation becomes particularly crucial. In [52], Ren et al. studied the joint resource allocation among sensor nodes, edge nodes, and cloud infrastructure, aiming to enhance the efficiency and real-time performance of ubiquitous computing systems through the optimized allocation of computing, storage, and communication resources. In [53], Li et al. proposed a resource allocation method for virtualized wireless sensor networks. This method combines wireless energy transmission and network slicing technologies, reducing energy consumption under performance constraints by optimizing frequency, time, and power. In [54], Ghanbari et al. explored resource allocation methods in the Internet of Things (IoT) and designed resource allocation strategies oriented towards cost, context, and efficiency awareness. In [55], Arif et al. summarized the resource efficiency optimization techniques in IoT applications, with a focus on analyzing resource allocation cases in industrial environments.

The key to transitioning from sensor-driven ubiquitous computing to embodied intelligence lies in exploring closed-loop systems in the later stages of ubiquitous computing. Traditional systems mainly follow a “perception-reporting-response” model, which is an open system that lacks feedback between perception and response. As a result, the system’s autonomy and adaptability are limited [56]. In recent years, researchers have started exploring closed-loop architectures in pervasive computing. These systems emphasize the feedback between perception, computation, and response. Their efforts have gradually shaped a preliminary framework that outlines the “perception-thinking-action” closed-loop characteristics of embodied intelligence [57]. For example, in smart home systems, some advanced solutions now enable real-time feedback between environmental perception and device control. Sensors collect data on device performance and adjust control strategies accordingly [58]. In industrial monitoring systems, researchers have explored feedback loops between equipment status perception and maintenance operations. Although these closed-loop explorations are still limited to simple scenarios and lack long-term autonomous learning and goal-driven capabilities, they show the feasibility and value of the “perception-thinking-action” model. This work lays an important foundation for the overall development of embodied intelligence [59].

**Figure 2:**
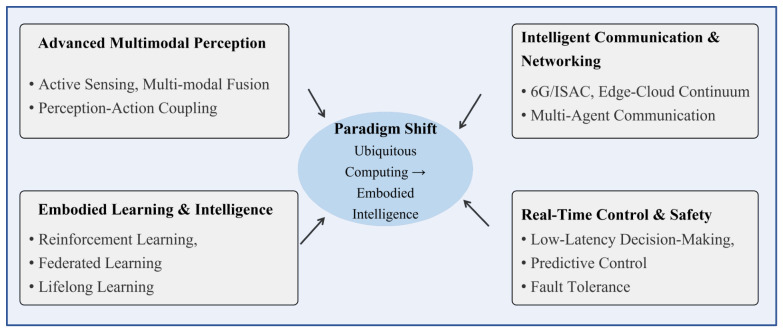
Key enabling technologies for the paradigm shift to embodied intelligence.

As shown in Figure 2, the paradigm shift from ubiquitous computing to embodied intelligence relies on four groups of enabling technologies: advanced multi-modal perception, intelligent communication & networking, embodied learning & intelligence, and real-time control & safety. Active sensing refers to goal-driven, adaptive perception rather than passive sampling. Integrated Sensing and Communication (ISAC) unifies perception and transmission to improve efficiency. Multi-agent communication ensures reliable coordination among distributed embodied agents. Together, these technologies enable closed-loop systems.

## 3. Key Research Directions of Sensor-Enabled Embedded & Ubiquitous Computing

Based on fundamental technologies, this section will delve into the key research directions in sensor-driven embedded and pervasive computing, with a particular focus on the aforementioned areas. Each research direction will be analyzed from its background, the latest progress, and optimization strategies, with particular emphasis on sensor integration and optimization.

### 3.1. Integration of Sensor-Driven Systems and Emerging Wireless Technologies

Driving embedded and pervasive computing systems with new wireless technologies can significantly enhance the performance of the entire network. This technological advancement can enhance the connectivity of various components within the system, reduce information transmission delay, and increase the bandwidth of information processing. Wireless communication technology is developing rapidly. Currently, 5G networks have been commercialized on a large scale, and the application scenarios of 6G have been determined and entered the stage of protocol standardization. With the advancement of wireless networks, the transmission of sensor data will become faster and more efficient. The ultra-reliability and low-latency communication capabilities gradually possessed by wireless networks will provide strong support for the application of sensors in fields such as health monitoring, industrial automation, and smart cities.

The integration of wireless networks and sensor networks makes the real-time transmission of a large amount of sensor data possible. In [60], Gururaj et al. proposed the use of wireless networks to transmit high-resolution images from monitoring sensors and real-time physiological data from wearable devices. The 5G-based distributed base station network architecture allocates independent communication resources for different types of sensor data through network-slicing [61]. In addition to wireless cellular networks, local area network technologies such as LoRa and NB-IoT can also be used to enhance the performance of wireless sensors. In [62], Pasolini et al. proposed to utilize LoRa and NB-IoT technologies to achieve high-efficiency and long-distance communication. On the other hand, researchers focus on optimizing wireless communication protocols to enhance compatibility and energy efficiency among different devices and networks. In [63], Li et al. studied the ECIoT framework and emphasized the management of wireless and computing resources. Building on these advancements, the interface between sensor networks and 5G/6G networks is designed across the protocol, network, and service layers. At the protocol layer, sensor data formats are adapted to 5G/6G QoS classes through standardized encapsulation and packetization. At the network layer, 5G/6G network slicing, flexible UL/DL configuration, and massive machine-type communications provide dedicated transmission channels for heterogeneous sensor data. Such a layered design guarantees efficient, reliable delivery of large-scale multi-source sensor data.

To date, most studies center on improving data transmission rates via wireless technologies, while real-time performance remains an under-explored research direction. While wireless networks excel at high-speed data delivery, they are optimized solely for throughput rather than the interactive coupling between software and physical entities. To sustain continuous interaction with dynamic environments, embedded intelligent systems must support real-time sensor acquisition alongside robust closed-loop channels for control commands and feedback signals. In [64], Klaudia et al. studied the data transmission design of robot systems, discussing how sensors can quickly process feedback and transmit it in real time, how to ensure that action instructions are transmitted promptly and accurately, and how to formulate strategies to enable the system to adjust its behavior or task execution.

### 3.2. Design, Analysis, and Optimization of Sensor-Driven Networks

Multiple sensors can constitute sensor networks. Hence, effective network design, data analysis, and resource optimization represent vital research directions. These investigations need to consider system coverage, connectivity, reliability, and energy efficiency, as well as the complexity of practical environments and limited node resources [65].

A wealth of existing studies have proposed targeted optimization schemes centered on the above performance indicators. In [66], Otero et al. leveraged the distributed sensing scheme to achieve wide-area, large-scale data collection via sensor networks. In [67], Liu et al. investigated the deployment methods of distributed sensors to reduce the risk of centralized network failures and thereby enhance the robustness of the system. In [68], the authors proposed to expand coverage by forwarding data through relay nodes in multi-hop sensor networks and reduce energy consumption through reasonable resource allocation. In [69], Liu et al. explored multiple metrics in distributed sensor networks, including coverage, connectivity, latency, and energy consumption. Considering the performance indicators and data transmission links, the research on sensor deployment, routing protocols, and load balancing algorithms constitutes the main content of the optimization research on wireless sensor networks. Researchers are working to optimize node layouts for maximum coverage and minimum energy use [70]. They’ve also developed energy-efficient routing protocols aimed at reducing both latency and energy consumption [71]. Load balancing efforts focus on evenly distributing data transmission and computing tasks [72]. Some studies have systematically examined the challenges faced by low-power microcontrollers. These studies also explore how artificial intelligence techniques can optimize performance and energy consumption in applications like smart homes and industrial automation [73].

Considering the movement of sensor nodes, the existing optimization algorithms are difficult to adapt well to the application of embodied agents in dynamic mobile environments [74]. In [75], Nian et al. proposed that the network needs to enhance performance by supporting low-latency collaboration between distributed sensors and embodied agents. Regrettably, the existing networks mainly focus on data collection and transmission, with less consideration given to collaboration with execution modules. Therefore, the network cannot form a closed loop of “perception-thinking-action” [76], which is crucial for embodied intelligence.

### 3.3. Sensor Data Analysis and Management

With sensor data volumes growing rapidly, robust data management has grown critical. Multi-modal sensing systems generate massive heterogeneous streams across pervasive and embedded computing platforms [77], whose core processing goal is to extract valuable insights while guaranteeing secure storage, transmission, and privacy protection [78]. Integrated IoT, edge, cloud, and AI architectures provide viable technical foundations to support low-latency real-time analysis and predictive decision-making [79]. Smart city deployments in Stockholm and Barcelona have validated such data-driven frameworks, including smart grid operation and environmental monitoring platforms [80]. Extensive practical research targets long-term health monitoring scenarios. The researchers designed embedded terminals to continuously capture physiological indicators for chronic diseases such as hypertension, diabetes, and depression [81]. Elgazzar et al. further proposed an intelligent IoT architecture that unifies sensing collection and AI analytics to enable real-time risk judgment, predictive maintenance, and user behavior profiling, though this integrated framework introduces inherent risks, including data heterogeneity and privacy leakage [82].

Privacy leakage stands as the most prominent bottleneck of sensor data applications, since raw sensing records contain sensitive geographic and personal physiological information prone to illegal exploitation. Current app consent mechanisms remain oversimplified and context-agnostic, lacking granular permission control for differentiated data disclosure. Two mainstream technical routes mitigate privacy risks while balancing operational efficiency. Saleh introduced the “Follow Me AI” paradigm, where intelligent agents manage authorized sensor data to optimize environmental regulation and predict user demands [83]. Perez et al. systematically summarized multi-layer privacy-preserving mechanisms tailored for pervasive sensor networks [84]. In addition, federated learning has emerged as a lightweight collaborative solution. The researchers enabled distributed model training across sensor nodes without exchanging raw private measurements [85]. Another related contribution comes from [86], which introduced the concept of “tangible privacy.” This concept emphasizes the clear communication of sensor status in IoT devices to enhance the privacy of bystanders. The research also explored security and privacy issues in smart cities and proposed future research directions, aiming to enhance data security, authentication, and device reliability. Furthermore, [87] has proposed a blockchain-based framework. This framework addresses the issues of data integrity, trust, and security in the IIoT. It ensures secure service delivery through pseudo-chain code and consensus protocols. In the 6G smart city environment, [88] discussed the security architecture required for these environments. This article mainly discussed how to provide reliable security guarantees in these environments.

To unlock the value of massive sensor datasets, scholars widely adopt machine learning pipelines for intelligent analysis, which can be categorized into supervised, unsupervised, and reinforcement learning paradigms. In [89], Li et al. utilized supervised learning algorithms to achieve industrial fault diagnosis and wearable sensor activity recognition. Supervised learning requires labels, which are often difficult to obtain and have high time and economic costs. For this reason, researchers have proposed unsupervised learning methods, which do not require labels and complete the classification and recognition of data by analyzing the features among the data. In [90], Samweslin et al. investigated these unsupervised learning methods, including K-means clustering and principal component analysis. Since unsupervised learning does not require labels, it can reduce the overhead of storage and transmission. Neither approach supports real-time environmental interaction. For this reason, researchers have proposed a reinforcement learning algorithm, which can better adapt to the environment through interaction. In [91], Ogbodo et al. utilized reinforcement learning algorithms for adaptive data acquisition and processing.

Complementary preprocessing technologies further improve data quality and transmission efficiency. To manage data more effectively, in [92], the authors proposed to utilize data compression techniques to reduce the size of sensor data. On the other hand, in [93], Li et al. proposed to integrate data from multiple sensors using data fusion methods to enhance the accuracy and completeness of the data. In [94], researchers proposed to utilize an edge cloud collaborative storage system to effectively balance real-time performance and data capacity. To achieve data quality control, in [95], the authors proposed an abnormal data checking method, which can detect and correct abnormal data to ensure the reliability of sensor data in subsequent analysis.

Despite the abundant above-mentioned data processing solutions, most existing algorithms operate in a passive trigger mode, incapable of supporting long-duration autonomous learning and lifelong knowledge accumulation [96]. This mismatch creates a critical gap for embodied intelligence systems, which require goal-driven continuous learning from historical sensing records and real-time interactive feedback. Accordingly, autonomous, efficient data collection and analysis pipelines remain an open research challenge worthy of further exploration [97].

### 3.4. Safety, Reliability, and Assurance in Sensor-Driven Networks

Sensor-driven embedded and ubiquitous computing systems are increasingly being applied in safety-critical and mission-critical fields. These fields include medical monitoring, industrial control, and intelligent transportation. Therefore, ensuring the security, reliability, and overall protection of these systems is especially important [98]. These systems face a variety of risks. These include perceptual deception (such as tampering with sensor data), data poisoning (such as injecting false data into the system), cyberattacks (such as eavesdropping and interference), and hardware failures. Any of these risks may trigger system breakdowns or even severe physical accidents [99].

For security architecture design, Al-Muhtadi et al. constructed a lightweight security framework that authenticates context senders and receivers to secure environmental sensing pipelines [100]. In [101], Mukherjee et al. studied the security architecture of the medical system, using advanced data processing techniques to predict health problems based on sensor data and health records while ensuring data security. Perez et al. further summarized universal privacy and security bottlenecks of pervasive sensor networks and outlined corresponding research directions [84]. Risk assessment identifies the safety risks of the sensor-driven system in different scenarios. Redundant design boosts the system’s reliability by adding backup sensor nodes. Real-time monitoring ensures the system operates continuously and allows for timely detection and resolution of faults [102].

Compared with traditional ubiquitous computing systems, embodied intelligence requires higher levels of security, reliability, and overall protection. Traditional systems mainly focus on data and network security threats. However, embodied intelligence directly interacts with the physical world, introducing new physical security risks. It is highly worth noting that attackers’ tampering with sensor data may lead to physical damage or injury. The reason is that incorrect data may cause the agent to make wrong decisions, thereby triggering dangerous actions. When agents possess stronger adaptability and autonomy, this kind of security threat becomes more unpredictable. In [103], Zhang et al. proposed a malicious attack on the system learning model. The existing security technologies mainly deal with static threats and lack the ability to address the challenges brought by flexibility in embodied intelligent scenarios. Meanwhile, in [104], Guan et al. analyzed the impact of fault-tolerant design on the physical interaction of sensor or component failures and proposed methods to deal with the constantly evolving threats in embodied intelligent systems.

In the upcoming five years, post-quantum reliability may become mandatory for embodied intelligent systems [105]. Due to their direct physical interaction, sensor data, perception results, multi-agent communication, and actuation commands must be protected by quantum-resistant cryptography. This work elaborates on embedding post-quantum algorithms to realize authentication, encryption, and integrity verification.

### 3.5. Machine Learning Techniques in Embedded and Ubiquitous Computing

According to [106], machine learning (ML) was driving sensor-based embedded and pervasive computing systems towards intelligence. However, deploying machine learning models in embedded systems is difficult, primarily due to the contradiction between the complexity of algorithms and the insufficiency of computing power, storage capacity, and energy. In [107], Pasricha et al. analyzed the impact of memory bandwidth and capacity on embedded systems. Recently, researchers’ attention has shifted to integrating machine learning with new technologies to empower sensor networks. In [108], Sodhro et al. explored the integration of machine learning and 6G technology and proposed a mobility management method based on machine learning for industrial environments. The research in [109] emphasized the application in the field of machine learning in medicine, introducing wearable equipment machine learning algorithms to solve the problem of environmental fast adaptability. In [110], Li et al. combined triboelectric nanogenerators with machine learning algorithms to design a novel gait recognition system. In [111], Chen et al. proposed a wearable sensor system driven by a personalized classifier to continuously monitor the user’s breathing behavior. For the cyber-physical sensing system of Industry 4.0, in [112], Eshmawi et al. proposed a data perception and coding scheme based on deep learning and meta-heuristic algorithms to reduce data communication volume. In [113], researchers proposed an elderly activity monitoring algorithm based on deep belief networks. For real-time medical applications, researchers have proposed the concept and system prototype of body area network in [114]. This system integrates technological innovations in cloud computing and machine learning, enabling more effective monitoring of patients’ health and support for remote management.

Researchers have proposed a variety of solutions for the deployment of machine learning models in resource-constrained environments. Firstly, researchers at [115] proposed that by integrating model compression techniques such as pruning and quantization, they designed a lightweight machine learning model to reduce computational and storage overhead. Among them, MobileNet and TinyCNN are two well-known efficient architectures. For instance, a lightweight CNN model was developed for sensor data classification, which reduced the model size by 70% while maintaining a high classification accuracy rate [116]. Secondly, distributed machine learning methods utilize edge computing and fog computing for hierarchical training and inference. Sensor nodes are responsible for data collection and preliminary model reasoning. Edge nodes perform data aggregation and local training. Cloud nodes optimize the global model to improve the accuracy of the model while ensuring real-time performance [117]. In addition, researchers often employ transfer learning methods to overcome data limitations in specific contexts. These methods improve the generalization ability of the model by transferring the knowledge of existing data to new scenarios [118]. Regarding privacy and security issues, [119] reviewed the application of sensing technology and federated learning in addressing privacy problems during the data collection process. This study also proposed new directions for future research in this field.

Currently, machine learning applications in pervasive computing mainly focus on perception and classification tasks, such as fault detection and activity recognition. However, they often overlook the “decision-making and action” aspect [120]. To demonstrate true intelligence, machine learning models that support goal-driven decision-making are necessary. These models allow the system to continuously learn from feedback and optimize action strategies as it interacts with the physical world [121]. In addition, existing lightweight models are generally designed for static tasks, and they struggle to adapt to dynamic environments that involve physical interactions [122]. According to the viewpoint of [123], the collaboration among distributed machine learning requires multiple agents to learn together and coordinate their actions, but there are many problems in the practical application process.

### 3.6. Spectrum Sensing and Sharing in Sensor-Driven Systems

When the number of sensor nodes increases, the demand for wireless communication will grow. At this time, the limited spectrum resources will restrict the development of embedded and pervasive computing systems. To address this challenge, in [124], researchers have proposed spectrum sensing and sharing technologies to enhance the efficiency of spectrum utilization, enabling sensor systems to share spectrum with other wireless networks.

In [125], researchers proposed to use spectrum sensing technology to monitor the occupation of spectrum resources in real time and identify idle frequency bands that can be used for sensor data transmission. In [126], researchers adopted multi-sensor spectrum fusion technology to enhance the accuracy and real-time performance of spectrum sensing. This technology can simultaneously detect the spectrum usage of multiple sensor nodes. In [127], Subray et al. proposed to utilize machine learning algorithms to enhance the capability of spectral sensing. There are mainly two types of spectrum sharing. The first is downlink sharing, where the sensor system and the licensed system can use the spectrum simultaneously without interference. The second is overlay sharing, where the sensor system uses the idle spectrum of the licensed system [128]. Recent research has focused on developing spectrum allocation algorithms. These algorithms aim to optimize resource allocation, maximize spectrum utilization, and minimize inter-system interference. For example, researchers have used particle swarm optimization to enhance channel allocation, thus improving the spectrum sharing effect [129]. In [130], Onumanyi et al. summarized the possibility of combining cognitive radio technology with LPWAN, and they also discussed various LPWAN technology details and emphasized the advantages of applying cognitive radio at the physical layer.

In [17], the researchers pointed out that embodied intelligence systems require more flexible spectrum sensing and sharing capabilities to adapt to dynamic mobile interaction scenarios. However, the current spectrum sensing and sharing technologies mainly focus on static sensor networks, which are difficult to meet the dynamic positioning and communication requirements of agents. On the other hand, the real-time interaction requirements in embodied intelligent systems are also related to task allocation. In the case of emergency operation commands, the network needs to prioritize the allocation of spectrum resources with higher priority. In [131], researchers analyzed whether the existing spectrum allocation algorithms could meet the requirements of real-time spectrum allocation. Furthermore, in [132], Navid et al. proposed effective interference management strategies to ensure reliable and uninterrupted real-time transmission.

### 3.7. Applications of Mobile Edge Computing and Fog Computing in Sensor Systems

Traditional centralized cloud computing fails to satisfy real-time actuation feedback due to excessive transmission delay and bandwidth pressure. Mobile edge computing (MEC) and fog computing deliver an essential distributed computing infrastructure to reconcile latency constraints, resource efficiency, and privacy protection for large-scale sensor networks. In recent years, research on combining MEC and fog computing with sensor systems has focused on three main areas. First, optimizing edge node deployment, which involves determining the optimal number and locations of edge nodes to maximize coverage and minimize latency [133]. Second, improving resource allocation between edge nodes and sensor nodes, including both computing and communication resources. Finally, edge-cloud collaborative computing takes full advantage of both edge and cloud nodes, boosting the efficiency of the whole system [134]. In [135], researchers summarized the technical paths for the integration of the IoT and edge computing, the main system architectures and key supports, and also discussed security and privacy issues regarding edge computing.

To further optimize the overall performance of such cloud-fog collaborative frameworks, in [136], researchers discussed the common problems of traditional cloud-based sensor data processing, including high latency and insufficient bandwidth. To overcome these challenges, MEC and fog computing bring computing resources closer to sensor nodes by deploying them at the network edge [137,138]. Additionally, ref. [139] examined how cloud and fog computing can work together to process data, particularly in medical applications, and showed that this collaborative approach is feasible. Researchers have applied methods like multi-agent deep reinforcement learning and mixed-integer linear programming to improve resource efficiency [140]. Compared to traditional cloud computing architectures, this distributed collaborative paradigm reduces end-to-end latency by 50% [141].

Although MEC and fog computing can enable real-time data processing in ubiquitous computing, they are better suited for “data center” processing and less effective for “action loop” processing [103]. Embodied intelligence requires integrating edge computing nodes with action modules to create a real-time feedback loop [142] between data processing (thinking) and action execution (doing). In [143], the authors investigated the dynamic access issue of mobile embodied agents during their movement and optimized the deployment of edge nodes. For embodied intelligence, edge-cloud collaborative computing must prioritize real-time decision-making and action feedback rather than merely focusing on large-scale data storage. According to the perspective of [144], the mainstream solutions mainly addressed the issues of data storage and complex model training, with less consideration given to real-time interaction requirements.

## 4. Transition from Sensor-Enabled Ubiquitous Computing to Embodied Intelligence: Core Upgrades and Key Technologies

Existing research pinpoints inadequate autonomy as the primary barrier of sensor-based ubiquitous systems, which needs to be overcome to realize EI.

### 4.1. Core Concepts of EI and Its Relationship with Sensor-Supported Ubiquitous Computing

An embodied intelligence system is a physically embedded intelligent system that integrates sensors, actuators, edge/cloud computing, wireless communication, and embodied learning to form a closed-loop architecture [145]. It directly interacts with the physical world in dynamic environments, supports goal-driven decision-making, autonomous adaptation, and continuous lifelong learning, and is widely applied to UAV swarms, mobile robots, autonomous vehicles, wearable agents, and industrial IoT scenarios under strict constraints of latency, energy, reliability, and security.

Embodied intelligence refers to the ability of an intelligent system to perceive the physical world through sensors and actuators. These systems can reason and make decisions based on their perception and existing knowledge, and then act on the environment. They continuously learn and adapt through interactive feedback [146]. Unlike traditional sensor-supported ubiquitous computing, which mainly focused on “perceiving the environment and responding,” embodied intelligence emphasized “interacting with and shaping the environment” through autonomous, goal-driven processes [147]. Table 2 summarizes the main differences between the two.

It should be noted that embodied intelligence does not completely replace sensor-supported ubiquitous computing but rather builds upon its foundation. Ubiquitous computing provides core technologies such as sensor integration, wireless communication, and edge computing, which serve as the foundation for embodied intelligence. This transformation unfolds gradually. A key milestone was the introduction of the “Action” module and the creation of a closed-loop “perception-thinking-action” system.

### 4.2. Sensor Role Upgrade: From Passive Data Collectors to Goal-Driven Perceptors

As shown in the “Sensor Roles” row of Table 2, in the transition from ubiquitous computing to embodied intelligence, sensors play a crucial role as they are responsible for the connection between the system and the physical world. Therefore, the role of sensors also needs to change, transforming from passive data collectors to active sensing elements [150]. This change is not merely a functional improvement, which marks a deeper transformation in the way sensors are integrated into the overall system architecture, and promotes the formation of a closed-loop perception-think-action paradigm [149], corresponding to the “System Model” row in Table 2.

In traditional ubiquitous computing, sensors typically operate at a fixed sampling rate or respond to external triggers, which is passive. In contrast, embodied intelligence requires sensors to be able to dynamically adjust their perception parameters, that is, the requirement of proactiveness. These include sampling frequency, sensing range, and data resolution. The adjustments depend on the current goals, task priorities, and environmental feedback [154]. For example, an embodied robot performing navigation tasks might increase the sampling frequency of its positioning and obstacle sensors when approaching complex environments. In open areas, it may reduce the sampling frequency, thus balancing energy efficiency and sensing accuracy [148]. This active perception relies on tight integration between the sensor modules and the system’s decision-making and learning components, which supports the “Core Objectives” row in Table 2.

Secondly, sensors have evolved from isolated perception to integrated perception-action coupling. In traditional ubiquitous computing systems, sensors are primarily responsible for data collection. They have relatively weak coupling with response or action modules. In embodied intelligence, sensors and actuators are deeply integrated. Together, they form tightly coupled perception-action pairs that enable real-time feedback between perception and execution [120]. This reflects the “Interaction Modes” difference shown in Table 2: ubiquitous computing relies on indirect, human-regulated interaction, while embodied intelligence enables direct, physical world-oriented interaction. For instance, when an embodied agent grabs an object, the force sensor continuously measures the contact force and sends this data to the decision-making module. The decision-making module then adjusts the actuator output to prevent slippage or damage [155].

Thirdly, perception has evolved from single-modal perception to multi-modal fusion [156]. Although traditional ubiquitous computing has gradually adopted multi-modal sensors, data fusion is mostly used to improve perception accuracy. In embodied intelligence, multi-modal fusion is specifically geared towards physical interaction. It combines vision, force sensing, touch, hearing, and positional sensing. This allows the system to capture a wide range of physical properties of the environment and objects, such as shape, hardness, location, and sound [31].

Finally, the function of the sensor has evolved from data-driven perception to knowledge-driven perception [151], which is clearly summarized in the In “intelligence Types” row of Table 2. In traditional ubiquitous computing, sensor data analysis primarily focuses on extracting task-specific features or patterns. In embodied intelligence, sensors contribute to building and continuously updating the world model, which is a structured representation of knowledge. This model includes environmental attributes, object behaviors, and feedback from past interactions [152]. The world model allows the system to predict the consequences of its actions while also supporting goal-driven decision-making and experiential learning. For instance, an embodied agent may learn from sensory feedback that dropping a fragile object, like glass, will cause it to break [153]. This lifelong learning capability is highlighted in the “Learning Capabilities” row of Table 2.

### 4.3. Key Technologies Driving the Transformation

Next, we will discuss the key technologies for transitioning to embodied intelligence. In [145], Long et al. discussed some of these capabilities of agents to support closed-loop interaction, autonomous learning, and adaptive behavior in the physical world. In [157], Latva-Aho et al. highlighted the key drivers of 6G. An overview of the IoT architecture and related technologies was discussed at [158]. However, these descriptions are not sufficient and have not been analyzed and summarized from the perspective of the true needs of embodied intelligence.

#### 4.3.1. Advanced Communication Technologies

In [159], Adil et al. summarized how to utilize wireless networks and software-defined networks to enhance the collaboration and efficiency of collaborative robotic medical systems. In [160], Banga et al. proposed an industrial network mobility management method that combines machine learning and 6G to enhance the quality of user experience. In [161], Mineeva et al. proposed a 6G network specifically for robot service delivery and proposed to evaluate the communication performance in robot services based on consumer quality metrics. In [88], for smart cities, the network performance was enhanced through 6G networks.

#### 4.3.2. Active Sensing and Multi-Modal Fusion Technologies

As mentioned earlier, active perception allows sensors to adjust their parameters dynamically. These adjustments depend on system goals and feedback from the environment. Multi-modal fusion brings together heterogeneous sensor data, including visual, force, and tactile signals. This supported more complex physical interactions [31]. Recent advances have led to the development of adaptive multi-modal fusion algorithms. These algorithms fine-tune the fusion weights based on task objectives. Additionally, a low-power active perception strategy for mobile embodied agents has been created. Moreover, real-time fusion technology driven by edge computing enables low-latency interactions [162]. The authors of [163] introduced a perception-based semantic multi-source heterogeneous data fusion method.

#### 4.3.3. Embodied Machine Learning

Embodied machine learning (EML) introduces a new paradigm to embodied intelligence, focusing on the tight coupling between the learning process and physical interaction [164]. Unlike traditional machine learning, which primarily deals with offline data analysis, EML allows the system to learn directly from the feedback generated by physical actions. This approach continuously enhances the system’s perception and action capabilities. Key technologies in EML include reinforcement learning for embodied interaction. In this method, the system learns action strategies through trial and error. Lifelong learning enables the system to accumulate long-term knowledge without catastrophic forgetting. Additionally, cross-scenario transfer learning allows the system to apply the knowledge gained from one scenario to new interaction contexts, thereby improving its performance [120]. The study [165] presented an optimization method for LWPN devices. This method uses an expected energy counting algorithm based on the multi-armed bandit model. By applying machine learning techniques, it improves energy efficiency and data packet transmission rates in IoT networks.

#### 4.3.4. Real-Time Closed-Loop Control Technology

Only by achieving real-time closed-loop control can the responsiveness of the perception-thinking-action loop be guaranteed. According to the description of perspective [166], closed-loop control schemes are the foundation of high-quality physical interaction. Firstly, by designing low-latency sensor data processing algorithms, it can help reduce the time interval between perception and decision-making. Then, by optimizing predictive control technology, the system can predict the outcome of actions and adjust strategies accordingly. Liu et al. presented a representative edge-computing-based real-time control mechanism that minimizes latency between decision output and physical actuation [29]. Finally, high-precision feedback control technology ensures that accurate actions are executed based on sensor input.

**Figure 3:**
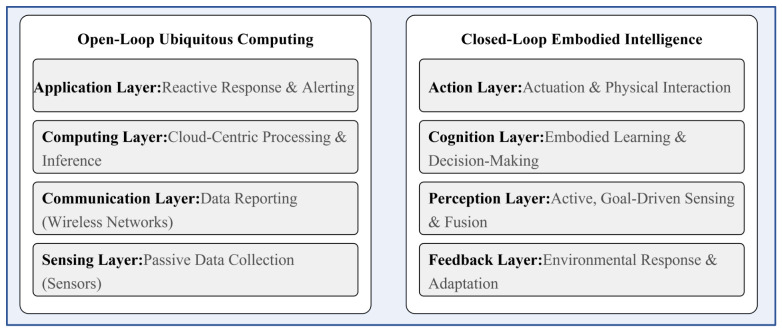
Architecture Comparison Between Open-Loop and Closed-Loop Embodied Intelligence.

As shown in Figure 3, the architecture comparison between open-loop and closed-loop embodied intelligence highlights the differences in control schemes and their impacts on system performance.

## 5. Application Scenarios and Case Studies: Ubiquitous Computing vs. Embodied Intelligence

This section introduces several representative application scenarios, including smart living, mobile and wearable systems, unmanned aerial vehicles (UAVs), etc. As shown in Table 3, a comparison of application scenarios between sensor-driven ubiquitous computing and embodied intelligence is provided. This table highlights the differences in how these two systems approach various domains, including smart homes, wearable health, and autonomous vehicles.

### 5.1. Smart Living Environments

In an intelligent living environment, achieving embodied intelligence is an important trend. In [167], Tang et al. pointed out that it is highly necessary to shift from the traditional “alert” model to an “autonomous service” system. Early sensor-supported ubiquitous computing solutions mainly relied on distributed sensors to monitor the environment and trigger abnormal state alerts or energy-saving suggestions by triggering preset rules or notifications [171]. Although these solutions can handle some daily tasks, they cannot adjust decision-making strategies based on long-term operational experience. In [172], Somov et al. designed wireless sensor networks, cloud computing, and artificial intelligence technologies to monitor and control plant and greenhouse environments. In [173], the authors applied low-power wireless technology to construct an energy-saving intelligent medical system. In [174], researchers summarized the development of medical sensors over the past 50 years. Furthermore, Javaid et al. also explored how these sensors are applied in the medical field and analyzed how IoT technology enhances their functionality.

With the introduction of embodied intelligence, the smart living environment is gradually evolving into an integrated system [175] that supports multi-device collaborative control, adaptive energy management, and proactive health support. Embodied agents (such as home service robots or intelligent infrastructure controllers) proactively coordinate various devices. These devices include lighting systems, HVAC equipment, household appliances, and medical or health sensors. The objective is to optimize the overall performance of the system under multiple objectives [176,177]. For instance, embodied controllers can adjust the operation strategies of devices based on occupancy patterns, user preferences, and real-time energy pricing. This helps to reduce energy consumption while ensuring user comfort [178]. Because embodied intelligence realizes the closed-loop system, it can better serve the health application [179]. This is because traditional systems only issue alarms when abnormal physiological signals are detected, while embodied intelligence systems can actively intervene. The embodied intelligence system can be adjusted according to environmental conditions to guide users in rehabilitation training or coordinate with caregivers and medical services [169].

### 5.2. Mobile and Wearable Systems

Embodied intelligence can also be applied to mobile and wearable devices, and there is a lot of research in this area. In [168], William et al. investigated how embodied intelligence addresses privacy issues through personalized modeling and real-time learning on terminal devices or edge nodes. In [180,181], for embedded agents such as smartwatches and wearable health monitoring devices, the authors investigated methods for continuously perceiving users’ activities, physical conditions, and interactions with the environment. Improve the adaptability of the system by designing an adaptive model. In [182], Tsai et al. studied how to reduce the reliance on cloud computing resources in wearable devices. Wearable devices are generally required for long-term use, so energy efficiency issues are extremely crucial. In [183], the author investigated how to prevent excessive battery consumption of the device. In [184], Geng et al. extended the device’s lifespan by dynamically adjusting the sensing frequency, model complexity, and computational load. In [185,186], considering privacy protection, the authors proposed to utilize federated learning to achieve a balance between data security and the long-term availability of mobile wearable devices.

### 5.3. Unmanned Aerial Vehicles

Embodied agents can also be used to solve the problems of autonomous vehicles and UAVs [187]. In [170], Xu et al. investigated multi-sensor perception capabilities and integrated planning and control strategies, as well as efficient collaboration with the surrounding infrastructure. Unlike traditional sensor-driven automation systems, embodied intelligence emphasizes closed-loop interaction with the environment and focuses on the ability to continuously adapt to uncertainties. In [188,189], the authors utilized the deep fusion of multi-source perception data to enhance the reliable decision-making of unmanned devices. Perceptual data usually comes from multiple sensor modalities, including vision, radar, lidar, inertial measurement units, and wireless communication signals. In [190], Liao et al. further explored the methods of integrating these data. In [191], Tang et al. proposed that the collaboration between drones and ground infrastructure has enhanced traffic efficiency and operational safety. Furthermore, in [192], Zhang et al. proposed to utilize ISAC to assist in the automated operation of autonomous vehicles and unmanned aerial vehicle systems. For synesthesia fusion, the authors of [193] have combined perception and communication within a unified framework, providing high-precision environmental perception and low-latency information exchange. Drones and drone swarms have become an emerging typical application of embodied intelligence in search and rescue, defense, and military scenarios. In search and rescue missions, drone swarms realize rapid environmental perception and regional search through multi-sensor fusion and multi-agent collaborative communication [194]. In defense and military applications, they rely on closed-loop perception–action architecture to complete autonomous navigation, target detection, and coordinated operations [195]. These scenarios highly match the characteristics of embodied intelligence, such as physical interaction, real-time closed-loop control, and multi-agent collaboration.

## 6. Standardization: Enablers for the Paradigm Transition

Standardization can address key issues such as interoperability, compatibility, and scalability when integrating various sensors, actuators, computing nodes, and communication technologies, thereby overcoming the fragmentation of sensor-driven systems and embodied intelligence technologies. This section will focus on the progress of standardization in the fields of sensors, pervasive computing, and embodied intelligence.

### 6.1. Key Standardization Areas for the Transition

Standardization is critical for ensuring interoperability, security, and closed-loop consistency in the transition from ubiquitous computing to embodied intelligence. Existing standards (e.g., IEEE 1451 for smart sensors, ISO 11784/11785, MQTT, and 5G NR) were primarily designed for passive sensing and open-loop data transmission, and thus cannot fully support active perception, real-time perception-action coupling, and lifelong learning required by embodied intelligence. Based on functional attributes and application objects, we classify existing mainstream standards for ubiquitous computing into four categories and clarify their original design orientations and applicable scenarios.

- Sensor and Actuator Standardization: This field focuses on unifying sensor interface specifications, data acquisition protocols, and actuator control interfaces. The existing ubiquitous computing sensor standards, such as the IEEE 1451 intelligent sensor interface standard and the ISO 11784/11785 RFID sensor standard, mainly target single-mode or static sensing requirements. However, embodied intelligence requires new standards. These standards must support active perception, multi-modal fusion, and tight integration between sensors and actuators. Dynamic perception parameter adjustment, such as adjusting the acquisition frequency and resolution, is of vital importance. Real-time feedback standards between sensors and actuators are also indispensable to achieve a closed-loop “perception-action” process. In addition, we also need updated standards for sensor calibration, accuracy, and energy efficiency. These standards must meet the requirements of mobile embodied agents (such as robots and wearable devices) in dynamic environments. For instance, the authors in [196,197] reviewed the standardization technologies for wireless communication, focusing on reliability, latency, scalability, and energy efficiency.
- Data: With the rapid growth of multi-modal sensing and interactive feedback data in embodied intelligence, there is an urgent need for unified data formats, storage, and processing standards. Traditional ubiquitous computing standards, such as MQTT for lightweight data transmission and JSON for data exchange, cannot meet the dynamic, real-time, and knowledge-driven data requirements in embodied intelligence. New standards must be set. These standards should define a unified format for multi-modal sensor data fusion, interactive feedback data, and world model data. This can achieve data sharing, processing, and knowledge reuse among different embodied agents and systems. Privacy and security standards are equally crucial, especially for sensitive data such as user behavior and environmental perception data. As embodied intelligent systems interact with humans more and more frequently, these standards will play a key role in protecting personal information. The hybrid architecture proposed by [198] aims to reduce energy consumption, enhance interoperability, and increase scalability in 5G systems and sustainable smart cities. In addition, ref. [199] proposed a standard terminal data acquisition and transmission solution. This solution addresses the challenges faced by the multi-functional beacon system in terms of data format, communication protocol, hardware standardization, and information security.
- Communication and Network Standardization: Current wireless communication standards serve distinct design objectives. For example, 5G NR with URLLC is specifically engineered to support ultra-low latency and ultra-high reliability for mission-critical control, whereas LoRaWAN targets low-power, wide-area, and low-data-rate monitoring applications and is not designed for low-latency or high-reliability interactive control. Although 5G NR/URLLC already provides strong low-latency and high-reliability capabilities [200], further extensions and optimizations are required to meet the unique demands of closed-loop embodied intelligence, such as synchronized sensing–action, real-time feedback, and multi-agent coordination. In contrast, LoRaWAN lacks intrinsic support for low-latency deterministic transmission and requires architectural enhancements to enable interactive physical interaction scenarios [201]. Several wireless communication standards underpin the IoT and sensor-driven networks. IEEE 802.15.4 forms the basis for low-rate wireless personal area networks, including ZigBee and Thread [202], while IEEE 802.11ah enables long-range, low-power Wi-Fi connectivity for IoT devices [203]. Wide-area low-power communication is supported by standards such as LoRaWAN, and cellular IoT standards, including NB-IoT and LTE-M, provide broader coverage [204]. To effectively support mobile embodied agents, new standards are needed that address dynamic network topology adjustments, multi-agent communication coordination, and spectrum resource allocation. Moreover, standards for embodied collaborative communication in edge clouds should be developed, including protocols for resource scheduling and mechanisms for knowledge synchronization.
- System and Architecture Standardization: The traditional hierarchical architecture standards for ubiquitous computing cannot meet the requirements of embodied intelligent systems. The new standard must clearly define the core components of the closed loop. It should outline the interaction mechanisms and interface protocols of these components. In addition, we need to establish standards for integrating embodied machine learning models with perception/action modules. These standards are crucial for the system’s functionality.

Then, we sort out mainstream technical standards involved in embodied intelligence application scenarios and analyze the capability gaps of existing standards in supporting embodied intelligent closed-loop perception, decision-making, and execution. The detailed analysis results are presented in Table 4.

### 6.2. Current Progress and Gaps

Significant progress has been made in the standardization of sensor-driven ubiquitous computing. Many international organizations, such as IEEE, ISO, and IEC, have developed mature standards. However, the standardization of embodied intelligence is still in its infancy. A considerable gap remains between the current standards and those required for the transition process. The existing standards have not yet fully addressed the needs of this transition.

- The current standards mainly focus on traditional ubiquitous computing. They cannot support the core features of embodied intelligence, such as active perception, closed-loop control, and autonomous learning. For instance, there is currently no unified standard for multi-modal sensor fusion, and no hard real-time standards exist to support closed-loop control feedback between sensors and actuators [205,206]. In this context, hard real-time refers to strictly bounded latency necessary for safety-critical physical interactions, while closed-loop control feedback denotes deterministic bidirectional signaling for perception–action coupling, rather than mere acknowledgments or asynchronous notifications.
- The standardization of embodied intelligence still faces fragmentation issues among different disciplines and application scenarios. There is no comprehensive framework covering the entire transition process. For instance, sensor standards mainly focus on hardware interfaces, while communication standards emphasize data transmission. The lack of coordination between the two hinders the seamless integration of the perception-thinking-action closed loop [207,208].
- The limited participation of industry and academia in the standardization of embodied intelligence has slowed progress in the formulation of new standards. The diversity of different application scenarios makes the standardization process more complicated. Scenarios such as smart homes, industrial robots, and healthcare have diverse demands, involving differences in sensors, communication technologies, and security measures [209].
- Outdated IoT standards fail to support long-term autonomous learning. WSNs are widely used in fields such as medical care, military surveillance, and public security. However, their application is limited by the insufficiency of node computing power and battery technology. The combination of WSN and cloud computing has great potential. However, to unleash this potential, standardized research is still required in architecture, network dynamics, and data management that is tailored for embodied intelligence. Existing standards, such as oneM2M, have established hierarchical architectures [210], common data models, and network abstraction layers for traditional ubiquitous computing and IoT systems. Similarly, standards including OCF, IEEE 1451, and ISO/IEC 30141 provide mature frameworks for device interoperability, data representation, and system integration. Nevertheless, these standards were designed for open-loop sensing and passive data reporting rather than lifelong learning in embodied intelligence. Furthermore, the authors of [211] put forward the vision of digital twins in the intelligent space. However, standardization challenges have hindered the wide adoption of digital twin technology. The studies of [212] also emphasized the lack of standardized knowledge representation and semantic interoperability in the IIoT. The absence of standardization significantly hinders system integration and efficiency.

As shown in Table 5, all existing mainstream standards are designed for the open-loop paradigm of ubiquitous computing. There are obvious capability gaps in dynamic perception, real-time coupling, interactive data processing, and closed-loop system operation, which are the core bottlenecks restricting the large-scale deployment of embodied intelligence.

## 7. Open Challenges and Future Research Directions

**Figure 4:**
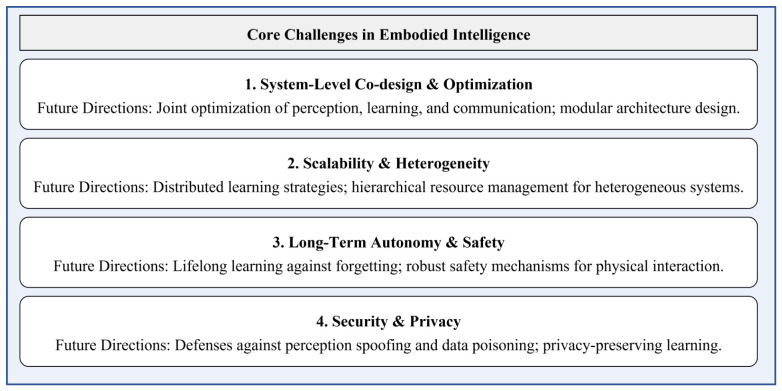
Key Research Challenges.

Despite significant progress in embodied intelligence, researchers still face several key challenges. As shown in Figure 4, these key research challenges are identified and discussed in terms of system-level design, scalability, long-term autonomy, and security [213]. This section outlines prospective research avenues centered on the construction of adaptive embodied intelligence.

### 7.1. System-Level Co-Design Challenges

Embodied intelligent systems will require an integrated common design approach that unifies perception, learning, and communication. Traditional designs usually optimize these modules separately, which limits the overall performance of the system. The co-design of embodied intelligence integrates sensor sampling strategies, data offloading mechanisms, and learning updates into an optimized framework. The key research directions include:

- Jointly optimize sensor sampling, communication scheduling, and edge inference within a unified perception-thinking-action closed loop. The goal is to cut end-to-end latency and power usage. Meanwhile, closed-loop stability, real-time response, and sensing precision must be preserved under tight resource limits.
- Build modular, reconfigurable architectures with unified hardware–software interfaces. Heterogeneous sensors, actuators, and learning models can be plugged in directly. No full-system rebuild is required, which greatly boosts scalability and maintainability.
- Develop hybrid simulation-physical test platforms and unified evaluation criteria. Core metrics include latency, energy efficiency, reliability, and safety. The platforms enable fair comparisons of co-design schemes under changing environments and fluctuating network loads.

### 7.2. Scalability and Heterogeneity

Embodied intelligence also needs to take into account the differences among various components in terms of perception accuracy, computing power, energy supply, and communication capabilities. To achieve overall optimality, hierarchical coordination and joint optimization are necessary. The key aspects that need to be considered for the unified optimization of the system include:

- Develop hierarchical control and dynamic resource scheduling algorithms. These algorithms allocate communication, computing, and sensing resources intelligently. Allocation rules depend on device capacity, task priority, interaction weight, and real-time network states to balance system performance and resource utilization.
- Design distributed robust optimization and online learning schemes. They adapt automatically to node faults, discontinuous connections, sensor bias, environment noise, and topology shifts. The design maintains steady, predictable performance in unstructured, dynamic physical scenes.
- Achieve deterministic, stable control quality. This design copes with system heterogeneity and scalability limits. It lays the foundation for large-scale, reliable deployment and safe physical interaction across diverse devices and scenarios.

### 7.3. Long-Term Autonomy and Lifelong Learning

To achieve continuous autonomous operation, embodied agents need to keep learning. They must adapt to dynamic and changing environments while maintaining stable performance. Embodied agents should also incorporate monitoring, auditing, and fault recovery mechanisms. These ensure long-term reliability. Key research directions include:

- Develop incremental and lifelong learning approaches. They support continuous knowledge accumulation, incremental model updates, and adaptive skill transfer. These methods alleviate catastrophic forgetting in dynamic open environments.
- Design safe constrained exploration for online reinforcement learning. The strategies embed physical limits and risk awareness. They secure stable and safe physical interaction during adaptation and model training.
- Build complete state management and fault recovery mechanisms. Functions include state recording, rollback, auditing, and error restoration. Such tools guarantee traceable decisions and long-term system resilience.

### 7.4. Security and Privacy in Physical Interaction

Different from general sensor network security, embodied intelligence introduces unique safety risks stemming from the closed-loop physical interaction. The key research directions include:

- Develop multi-modal consistency verification and anomaly detection modules. They cross-check data from multiple sensors and defend against spoofing, deception, and data tampering attacks.
- Construct robust learning and control frameworks equipped with adversarial defense. The systems maintain safety, constraints, and stability under noise, malicious perturbations, or partial sensing.
- Explore privacy-preserving distributed learning schemes such as federated learning. They protect sensitive sensor data and user information without sacrificing real-time performance or control precision.
- Build a unified evaluation system with quantitative indicators. It comprehensively measures adaptability, robustness, physical safety, and security in various practical scenarios.

## 8. Conclusions

In this paper, we review the evolution from sensor-driven ubiquitous computing to embodied intelligence, with a focus on key technologies, challenges, and emerging research trends. Firstly, we explored the basic system architecture and explained how the integration of sensors, actuators, and computing resources enables the system to have higher autonomy and interactivity compared to traditional ubiquitous computing. Then, we discussed the key technologies, ranging from sensor-driven embedded systems to embodied agents. Then, we summarized the research progress of embodied intelligence in fields such as smart living environments, mobile and wearable systems, self-driving cars, and unmanned aerial vehicles. Finally, we summarized the standardization work and future research directions.

## Figures and Tables

**Table 1:** From Sensor-Driven Ubiquitous Computing to Embodied Intelligence: A Comparison of Goals, Loops, and Constraints.

Dimensions	Sensor-Driven Ubiquitous Computing (Typical)	Embodied Intelligence Systems (Goal-Oriented)
Goals	Inference/recognition accuracy, timely alerts [2]	Long-term rewards (task completion, cost, and risk) [9]
Decision Loops	Weak loops (reactive) [1]	Strong loops (perception-thinking-action)
Learning Modes	Primarily offline training with regular updates [6]	Online/lifelong learning and continuous adaptation [14]
Architecture	Layered pipelines, cloud-centric [3]	Closed-loop architectures, multi-agent architectures, edge-cloud collaboration [17]
Key Constraints	Data acquisition/transmission energy and coverage [4]	Latency determinism, reliability, security and privacy compliance [16]
Failure Consequences	Informational level (false/missed reports) [7]	Physical level (security incidents, mission failure) [15]

**Table 2:** Key Differences Between Sensor-Supported Ubiquitous Computing and Embodied Intelligence.

Key Dimensions	Sensor-Supported Ubiquitous Computing	Embodied Intelligence
Core Objectives	Perceiving the environment and providing passive responses [1]	Interacting with the environment to achieve goal-driven autonomous adaptation [9,148]
System Model	Open loop: perception → reporting → response [9]	Closed loop: perception → thinking → action → feedback → learning [149]
Sensor Roles	Passive data collectors, peripheral or core components [29]	Active, goal-driven sensors integrated with the Action module [150]
Intelligence Types	Data-driven, task-specific [2]	Knowledge-driven, generalizable autonomous intelligence [151]
Interaction Modes	Indirect, human-regulated [3]	Direct, physical world-oriented [120]
Learning Capabilities	Static, primarily offline learning [6]	Dynamic, online, lifelong learning [14,152,153]

**Table 3:** Application Scenario Comparison: Ubiquitous Computing vs. Embodied Intelligence.

Application Domain	Sensor-Driven Ubiquitous Computing	Embodied Intelligence Systems
Smart Living Environments	Environmental monitoring, rule-based automation	Autonomous multi-device collaboration, proactive service [167]
Mobile and Wearable Systems	Physiological data logging, activity recognition	Personalized health modeling, real-time intervention [168,169]
Unmanned Aerial Vehicles	Multi-sensor data fusion, environment perception [170]	Closed-loop control, adaptive navigation, interaction

**Table 4:** Capability Gaps Analysis of Existing Mainstream Standards for Embodied Intelligence.

Standard Category	Typical & Standards	Capabilities Supported by Existing & Standards	Unmet Requirements for Embodied Intelligence (Specific Gaps)
Sensor & Actuator	IEEE 1451, ISO 11784/11785	Static sensor interface, single-mode data collection, offline calibration	1. No specifications for dynamic adjustment of active sensing parameters; 2. Absence of hard real-time interfaces for sensor-actuator closed-loop feedback; 3. Lack of dynamic calibration rules for mobile sensors in dynamic scenarios.
Data & Application	MQTT, JSON, oneM2M, OCF	Lightweight data transmission, static data format, basic device data interoperability	1. No unified specification for multi-modal fusion and physical interaction data; 2. Deficient standardized representation for world models and lifelong learning knowledge; 3. Missing dynamic data authorization and privacy computing specifications for interactive scenarios.
Wireless & Network	5G NR, LoRaWAN, NB-IoT, IEEE 802.15.4	Ultra-low latency, ultra-high reliability transmission	1. No dedicated protocol for sensing-action signal synchronous linkage; 2. Absent standards for multi-agent collaboration and knowledge synchronization. 3. Incapable of hard real-time deterministic transmission for closed-loop control; 4. Lack of dynamic spectrum scheduling for mobile embodied agents.
System Architecture	oneM2M, ISO/IEC 30141	Hierarchical open-loop architecture, open-loop data service	1. No interface standards for perception-thinking-action-feedback closed-loop architecture; 2. Missing specifications for lifelong learning model iteration and version management; 3. Deficient unified safety, defense and fault tolerance standards for physical interaction systems.

**Table 5:** Capability Gap Between Existing Standards and Embodied Intelligence.

Layer	Existing Standards & Core Capabilities	Standard Requirements of Embodied Intelligence	Capability Gap
Device Layer	Passive data collection; static calibration; fixed hardware interfaces	Dynamic active perception; sensor-actuator hard real-time coupling; mobile dynamic calibration	Lack of dynamic parameter adjustment and real-time linkage specifications
Data Layer	Open-loop data transmission; static data modeling; basic interoperability	Multi-modal fusion format; world model representation; privacy-preserving collaborative rules	No unified standard for interactive data and knowledge modeling
Network Layer	Static spectrum allocation; single-point data transmission; partial low-latency service	Perception-action synchronous transmission; multi-agent communication; deterministic hard real-time	Unable to support dynamic networking and closed-loop signal synchronization
System Layer	Hierarchical open-loop architecture; passive reporting logic	Closed-loop interface; lifelong learning iteration; physical safety & multi-agent collaboration	No specifications for closed-loop systems and autonomous learning

## Data Availability

No new experimental data were generated in this paper.
